# Underestimated and Overlooked Factors in PBC Progression: Bacterial and Fungal Infections

**DOI:** 10.3390/ijms27062766

**Published:** 2026-03-18

**Authors:** Yaxin Zhu, Sumeng Li, Shiqi Li, Yichen Wang, Yanqin Du, Xin Zheng, Jun Wu

**Affiliations:** Department of Infectious Diseases, Union Hospital, Tongji Medical College, Huazhong University of Science and Technology, Wuhan 430022, China; zyx1419415725@163.com (Y.Z.); sumengli618@163.com (S.L.); lishiqi9767@163.com (S.L.); wangycka@163.com (Y.W.); yanqindu@163.com (Y.D.); xinz@hust.edu.cn (X.Z.)

**Keywords:** primary biliary cholangitis, pathogenesis, bacterial infection, fungal infection

## Abstract

Primary biliary cholangitis (PBC) is a chronic autoimmune liver disease characterized by cholestasis, which can progress to end-stage liver disease and even hepatocellular carcinoma. Its onset is typically triggered by complex interactions between genetic and environmental factors. In recent years, epidemiological and mechanistic studies have highlighted bacterial and fungal infections as potential key environmental factors in PBC pathogenesis. Bacteria may be associated with PBC autoimmunity through mechanisms such as molecular mimicry. Gut microbiota dysbiosis has been linked to aberrant immune recognition, altered metabolites, and intestinal barrier disruption, which may contribute to the aggravation of liver injury. Case reports of fungal infections suggest an association with poor prognosis in PBC, although the underlying mechanisms remain to be elucidated. This review systematically summarizes existing clinical epidemiological data, microbiome association studies, and mechanistic evidence; synthesizes the possible molecular mechanisms linking bacterial infections to PBC development and progression; discusses the potential role of the gut microbiota in PBC progression; and analyzes the possible molecular mechanisms underlying the poor prognosis associated with fungal infections in PBC. This study aims to provide valuable insights for developing optimal prevention, diagnosis, and treatment strategies targeting bacterial and fungal infections in PBC.

## 1. Introduction

Primary biliary cholangitis (PBC) is a chronic cholestatic liver disease that primarily affects middle-aged and elderly women. Its hallmark pathological feature is progressive, non-suppurative, destructive inflammation of the small bile ducts, ultimately leading to biliary cirrhosis [1]. Clinically, patients typically present with fatigue, pruritus, and other cholestatic symptoms. Notably, the incidence of PBC has risen significantly in high-latitude regions of the Northern Hemisphere, including the United Kingdom and Italy [2]. A similar trend has emerged in Asia, with reported incidence rates in China ranging from 1.8 to 4.2 cases per 100,000 individuals. Furthermore, the proportion of male PBC patients is increasing, and elderly patients often exhibit a more severe disease phenotype and poorer prognosis [3].

The occurrence of PBC is closely associated with genetic, immune, metabolic, and environmental factors. Smoking [4], repeated exposure to certain cosmetics, and microbial infections are common environmental factors [5]. Microbial exposure is increasingly recognized as a key environmental determinant of PBC progression. In a case–control study from the UK, PBC patients exhibited significant differences from controls in multiple environmental exposures and lifestyle factors, including a history of urinary tract infections and herpes zoster, suggesting that these factors may be associated with an increased risk of PBC development [6].

When patients with PBC develop concomitant infections, cholestatic markers (such as elevated alkaline phosphatase [ALP]/γ-glutamyl transpeptidase [GGT] levels and hyperbilirubinemia) may be confounded by infection-related inflammatory responses. Systemic infections can precipitate acute decompensation in chronic liver disease, accelerate cholangitic injury, and significantly reduce long-term survival. Moreover, microbial infections increase therapeutic complexity, as antibiotics may interact with ursodeoxycholic acid, obeticholic acid, or immunomodulatory agents used to manage PBC. This necessitates dose reduction or treatment discontinuation, which can potentially compromise disease control. Thus, while clinicians may focus on anti-infection treatment, they underestimate the role of infections in PBC progression. Therefore, investigating microbial infections in the pathogenesis of PBC and its progression is crucial, offering a more rigorous perspective on their role in the disease.

Among the microbial taxa associated with PBC, bacteria and fungi are most closely linked to the disease’s pathophysiology. Gut microbiota translocate through a compromised intestinal barrier, persistently triggering chronic immune activation. Long-term immunosuppressive therapy for refractory disease further expands the niche for opportunistic bacteria and fungi. In recent years, a few reports have suggested an association between human β-retrovirus (HBRV) and PBC. However, subsequent studies have been unable to replicate these positive findings, raising suspicions of laboratory contamination. Currently, the absence of consistent seroepidemiological data and the lack of a recognized animal or cellular model have kept the viral hypothesis confined to the realm of “controversy,” preventing it from becoming a sustained research focus [7,8]. Therefore, our analysis focuses on the role of bacterial and fungal pathogens in the pathogenesis and progression of PBC.

## 2. Immunopathogenic Mechanisms of PBC

The primary pathogenesis of primary biliary cholangitis varies across different disease stages, ranging from cholangitis and liver fibrosis to liver failure/hepatocellular carcinoma. During the early cholangitis phase, specific autoantibodies—particularly anti-mitochondrial antibodies (AMAs)—bind to antigens on the mitochondrial membranes of bile duct epithelial cells. This binding triggers an immune response that ultimately damages bile duct epithelial cells (BECs) [9]. During this process, natural killer cells directly attack BECs, while macrophages phagocytose antigens and secrete pro-inflammatory cytokines, thereby activating lymphocytes [10]. Additionally, CD4^+^ helper T cells stimulate CD8^+^ cytotoxic T cells to attack the hepatobiliary ducts and promote B cells to secrete autoantibodies, further exacerbating tissue damage [11]. Additionally, cholestasis in primary biliary cholangitis is associated with impaired formation of the apical membrane bicarbonate “umbrella” in bile duct epithelial cells [12], genetic regulation, and abnormal repair processes following bile duct epithelial damage [13]. The primary underlying cause is the downregulation of the key anion exchanger AE2, a process regulated by the miR-506/AE2/sAC axis [14]. In the early stages of disease, the inflammatory response is primarily governed by genetic factors. Signaling pathways such as IKK/NFκB, JAK/STAT, and MAPK influence the pathological process by regulating the transcription of pro-inflammatory genes, the differentiation of immune cells, and the apoptosis of cholangiocytes [15,16,17]. Additionally, the cGAS-STING pathway-mediated senescence phenotype in biliary epithelial cells [18] and dysregulated Kupffer cell autophagy leading to CD8^+^ T-cell activation [19] provide new insights into PBC pathogenesis. These pathways have also been implicated in various other liver diseases, including non-alcoholic fatty liver disease, alcohol-associated liver disease, and viral hepatitis. However, recent studies have revealed several molecular mechanisms with high specificity for PBC. For example, IL-15Rα^+^ B cells recruit TRM cells via the CCL3-CCR5 axis, which, in turn, cleave GSDMB through GZMA to induce pyroptosis in biliary epithelial cells [20]. This represents a bile duct injury mechanism that is first elucidated in PBC, although its regulatory role in other chronic liver diseases remains to be clarified.

As PBC progresses to the hepatic fibrosis stage, stalled bile acids bind to sphingosine-1-phosphate receptor 2 (S1PR2) on hepatic stellate cells (HSCs), thereby activating pathways such as p38 mitogen-activated protein kinase (MAPK) and Yes-associated protein (YAP) [21]. Inflammatory cytokines that are upregulated during cholestasis, including interleukin-17 (IL-17) and tumor necrosis factor-alpha (TNF-α), can also directly activate HSCs [22]. Additionally, HSCs internalize exosomes containing specific non-coding RNAs from BECs, a process that further enhances HSC activation and ultimately leads to PBC-associated cirrhosis [23]. Another key regulatory factor is the junctional adhesion molecule (JCAD). JCAD binds to large tumor suppressor 1/2 (LATS1/2) and inhibits its kinase activity, resulting in reduced phosphorylation of YAP. Unphosphorylated YAP accumulates in the cell nucleus, activates downstream targets, and promotes HSC proliferation, activation, and extracellular matrix synthesis [24,25].

A growing body of evidence indicates a crucial link between advanced PBC and an increased risk of hepatocellular carcinoma (HCC) [26]. A key factor is the shift in Smad protein phosphorylation from the tumor-suppressing pSmad3C pathway to the oncogenic pSmad3L pathway [27]. The incidence of HCC is higher in male PBC patients than in female patients [28]. This disparity may be attributed to factors such as estrogen signaling, X-linked gene regulation, and activation of the PI3K/AKT/mTOR pathway [29].

## 3. Infection Susceptibility in Patients with PBC and Potential Mechanisms

Although no large-scale clinical cohort studies have definitively established the incidence of systemic infections in PBC patients, research into its molecular mechanisms has identified a potential risk of infection. In the early stages of the disease, PBC centers on an autoimmune response targeting small bile ducts. Its immunopathological features include dual dysregulation of innate and adaptive immunity, such as enhanced bile duct epithelial cell responses to microbe-associated molecular patterns, imbalanced T-cell subsets, and abnormal B-cell activation, leading to high-titer anti-mitochondrial antibodies. This abnormal immune regulation not only drives bile duct injury but may also weaken the body’s effective defense against genuine pathogens [30]. Concurrently, bile acid metabolism disorders accompanying cholestasis further disrupt gut–liver axis homeostasis. Through persistent inflammatory stimulation and exposure to microbial-associated molecules, this further amplifies immune activation in the liver [31]. Recent studies further indicate that hepatic macrophages in PBC are persistently activated and functionally impaired. Their chronic inflammatory response, mediated through pathways such as LPS-TLR, promotes bile duct injury while potentially exhibiting relative deficiencies in phagocytic and bactericidal capabilities. This results in a form of ‘functional immunodeficiency,’ even in the non-cirrhotic stage [32].

As PBC progresses to the stages of liver fibrosis and cirrhosis, patients with the latter commonly exhibit reduced complement synthesis, impaired innate immune cell function, leukopenia caused by splenomegaly, and a state of immune paralysis induced by chronic endotoxin exposure. These changes collectively weaken the body’s ability to clear pathogens such as bacteria and fungi, significantly increasing the risk of systemic infections and opportunistic infections [33].

The increased susceptibility to PBC infection stems from the early convergence of cholestasis and immune-mediated mechanisms, which later overlap with factors associated with cirrhosis. Of note, the above mechanisms are primarily derived from animal model studies and in vitro experiments, and their direct validation in PBC patients requires confirmation through large-scale clinical studies. As the rising incidence of PBC leads to an expanding high-risk population, the corresponding clinical burden of associated infections also increases, underscoring the necessity for systematic research in this field. In the following sections, we will focus on elucidating the mechanisms by which bacterial and fungal infections influence the progression of PBC.

## 4. Pathogenesis of Bacterial Infection in PBC

### 4.1. Bacterial Infections in PBC

Multiple studies have provided important biological and immunological evidence to explain the complex, multifaceted interactions between bacterial infections and PBC pathogenesis.

Early, small-scale case–control studies failed to establish a clear association between PBC and infection [34]. However, with increased sample sizes and subsequent validation studies, a significant association between urinary tract infections (UTIs) and the onset of PBC has been firmly established. A meta-analysis of seven case–control studies involving 4197 PBC patients and 21,237 controls reported that a history of UTIs was associated with an increased risk of PBC (pooled OR = 1.50, 95% CI: 1.26–1.77, *p* < 0.01), with consistent findings across North American (OR = 1.34, 95% CI: 1.23–1.46) and European populations (OR = 1.79, 95% CI: 1.37–2.33) [35]. Another meta-analysis confirmed this association (OR = 2.02, 95% CI: 1.40–2.65) [36]. Large case–control studies have reported similar effect sizes, with odds ratios ranging from 2.0 to 2.7 [5,37,38]. *Escherichia coli* (*E. coli*) has been identified as a key bacterium in this type of UTI; its pathogenic mechanism is mediated by molecular mimicry and cross-immunoreactions. This provides concrete mechanistic insights into how infection triggers or exacerbates autoimmune responses in PBC [39,40]. The E2 subunit of the pyruvate dehydrogenase complex, derived from *Escherichia coli* (PDC-E2), exhibits structural homology with human PDC-E2. The immune response elicited by *E. coli* PDC-E2 peptides (31–44/134–147/235–248) can induce persistent cross-reactivity against human PDC-E2 (163–176) [41]. This mechanism is supported by multiple lines of evidence, such as *Escherichia coli* infection in (NOD). B6 Idd10/Idd18 mice spontaneously induce AMA and cholangitis [42], and nearly identical T-cell receptor beta (TCRβ) repertoires exist between *E. coli*-specific and human PDC-E2-specific T cells [43]. Bauer et al. observed that the positivity rate of anti-*E. coli* antibodies reached 50% (21/42) in AMA-negative PBC patients and 74% (54/73) in AMA-positive PBC patients, both of which are significantly higher than the 28% (15/54) observed in healthy controls (*p* < 0.001). Furthermore, in vitro experiments demonstrated that *E. coli* peptides significantly inhibit the binding of antibodies specific to PDC-E2 [44]. Overall, these findings suggest that cross-immunological responses induced by *E. coli* infection are not only associated with AMA production but may also independently contribute to the pathogenesis of PBC (Table 1).

In addition to *Escherichia coli*, several other bacteria are implicated in the pathogenesis of PBC. A Gram-negative α-proteobacterium, *Novosphingobium aromaticivorans*, is another pathogen associated with PBC and exhibits high amino acid sequence homology with human PDC-E2 [45]. Infection of C57BL/6, NOD, and SJL mice with this bacterium induces the production of AMA, leading to bile duct injury and granuloma formation. Natural killer T (NKT) cells expressing CD1 recognize the cell wall sphingolipids of *Novosphingobium aromaticivorans* and secrete Th1/Th2 cytokines that drive downstream immune responses [46]. Repeated inoculation of C57BL/6 mice with *Intermediate streptococci* induces PBC-like chronic non-suppurative destructive cholangitis and elevates anti-gp210 antibody titers, consistent with findings in BALB/c mice [47]. Furthermore, 16S rRNA sequencing of liver tissue from PBC patients indicates that *Propionibacterium acnes* is involved in granuloma formation, although the underlying mechanisms remain to be elucidated [48] (Table 1).

Beyond the direct pathogenic effects of these bacteria, the regulation of immune cell subsets is crucial to the progression of PBC, with follicular helper T cells (Tfh cells) serving as a key mediator. Tfh cells (CD4^+^CXCR5^+^ T cells) exert their effects through molecules, including programmed cell death protein 1 (PD-1), CD40 ligand (CD40L), inducible co-stimulatory molecule (ICOS), and interleukin (IL-6, IL-10, and IL-21). These molecules promote B-cell differentiation into antibody-secreting cells [49]. Notably, stimulation with Toll-like receptor (TLR) ligands (e.g., Pam3CSK4, Poly(I:C), lipopolysaccharide (LPS), and CpG) or whole bacterial cells (e.g., *Escherichia coli*, *Novosphingobium aromaticivorans*, and *Mycobacterium gordonae*) can induce CD4^+^CXCR5^+^ T cells to secrete interferon-γ (IFN-γ), IL-17, and IL-21. This Tfh cell-mediated cytokine response further amplifies the autoimmune cascade, ultimately accelerating the progression of PBC (Table 1).

**Table 1:** Pathogenesis of PBC induced by different bacterial infections.

Pathogen Type	Research Method	Conclusion	Level of Evidence	Explanation of Evidence Levels	Significance	Citation
*Escherichia coli*	Animal model (Non-Obese Diabetic (NOD). B6 Idd10/Idd18 Mice)	*Escherichia coli* infection can induce PBC and produce AMA in susceptible mice.	IV	Mechanistic research: It demonstrates pathogenic potential under artificial conditions but cannot fully simulate the complexity of human diseases.	Specific pathogen infection can serve as an environmental trigger to initiate autoimmune diseases in genetically susceptible individuals.	[42]
	Clinical Study (T-Cell Receptor High-Throughput Sequencing)	In patients with PBC, memory T cells enriched for *E. coli* antigen recognition and clonal expansion are present.	III	Case–control study: It suggests that immune memory is associated with the disease, but the causal relationship cannot be determined.	Infection selects and amplifies pathogenic T-cell clones capable of cross-reacting with self-antigens through “molecular mimicry.” These clones may drive long-term autoimmunity.	[43]
	Molecular Immunology (Protein Purification and Antibody Reaction Analysis)	A lipoylated protein highly similar in structure to the PBC autoantigen PDC-E2 was identified from *Escherichia coli*.	IV	Mechanistic research: It provides a structural basis for the “molecular mimicry” theory, but its pathogenicity needs to be verified in vivo.	This study provides the most direct molecular target evidence for the “molecular mimicry” theory, elucidating the structural basis for cross-immunoreactions.	[41]
*Novosphingobium aromaticivorans*	Serology and Immunology (Antibody Reactivity Testing)	Serum from patients with PBC exhibits significantly heightened reactivity to the common environmental bacterium *Novosphingobium aromaticivorans*.	III	Case–control study: Shows the association between environmental exposure and disease.	Widespread exposure to environmental microorganisms may represent a significant risk factor for PBC induction.	[45]
	Animal Models (Comparison of Germ-Free Mice and Conventional Mice)	In a germ-free environment, cholangitis in PBC-model mice is alleviated; however, disease is induced following re-colonization with commensal bacteria, including *Novosphingobium aromaticivorans*.	IV	Mechanistic research: It has been proven that the symbiotic microbiota is a necessary condition for breaking immune tolerance, but the independent effects of specific bacterial species still need to be verified.	The commensal microbiota is not merely an accompanying phenomenon but a necessary condition for breaking immune tolerance and triggering autoimmunity.	[47]
*Lactobacillus*	Serology (Immunoblotting and Antibody Subtyping)	Serum from PBC patients can simultaneously recognize mitochondrial autoantigens and homologous proteins from *Lactobacillus*, primarily IgG3 antibodies.	III	Case–control study: It suggests the existence of cross-reactions, but more evidence is needed to support its pathogenic role.	It has been demonstrated that immune responses against commensal bacteria (such as *Lactobacillus*) can induce autoimmunity through cross-reactivity. The IgG3 subtype indicates that this represents a strong T-cell-dependent immune response.	[50]
*Propionibacterium acnes*	Clinical Pathology (Liver Tissue PCR Detection)	DNA of *Propionibacterium acnes* was detected in liver tissue (within granulomas) from PBC patients.	V	Descriptive research: It provides evidence of existence and offers preliminary clues for the hypothesis of “bacterial translocation to the liver”, but the causal relationship remains unclear.	The bacteria or their components may migrate from the gut to the liver, acting as an in situ antigen to directly trigger local immune responses and granuloma formation.	[48]
Multiple bacteria	In Vitro Cellular Experiments (Antigen Stimulation and Cytokine Assays); Clinical Serology (High-Throughput Antibody Microarray)	Follicular helper T cells from PBC patients exhibit enhanced reactivity to multiple bacterial antigens and produce increased levels of IL-21. Serums from PBC patients exhibit a specific antibody profile targeting multiple bacterial antigens.	IV; III	Mechanistic research: It reveals that patients with PBC have a “hyper-responsive” state to T-cell subsets, but this is an in vitro phenomenon. Case–control study: It suggests that extensive bacterial exposure is associated with abnormal immune responses.	The key helper T-cell subsets in PBC patients are in a “hyperactive” state, enabling them to more effectively assist B cells in producing autoantibodies and accelerating disease progression.	[44,49]

It should be noted that the above evidence derives from studies of varying levels, each with different weight: the association between *E. coli* and PBC is supported by multiple lines of evidence, including animal models, T-cell receptor repertoire analysis, and molecular mimicry mechanisms, making it the most clearly implicated candidate pathogen. Evidence for *Novosphingobium aromaticivorans* is derived primarily from animal experiments and serological associations; however, its direct pathogenic role in humans requires further validation. The detection of *Propionibacterium acnes* in liver tissue by PCR suggests its possible involvement in local granuloma formation, but a causal relationship has not been established. Therefore, it is inaccurate to regard these pathogens as equivalent “causative agents” of PBC, and future research should focus on establishing clearer causal links. Beyond the pathogenic roles of these specific bacteria in PBC progression, the complex and dynamic gut microbiota also plays a crucial and unique role in modulating disease pathogenesis.

### 4.2. Gut Microbiota and PBC Progression

#### 4.2.1. Changes in the Gut Microbiota of PBC Patients

Compared to healthy individuals, the gut microbiota of PBC patients exhibits distinct characteristics: α-diversity (measuring species richness and evenness within a single sample or environment) is typically reduced, and microbial dysbiosis is characterized by decreased levels of *Firmicutes* and increased levels of *Proteobacteria*. At the genus level, multiple studies have consistently reported enrichment of opportunistic pathogens. Lv et al. reported a 10- to 30-fold increase in *Veillonella*, approximately 5-fold increases in both *Streptococcus* and *Lactobacillus*, and a less than 5-fold increase in *Enterobacteriaceae*. Additionally, this study identified significant enrichment of several opportunistic pathogens rarely found in healthy individuals, including *Paraprevotella clara* (596-fold increase), *Megasphaera micronuciformis* (73-fold increase), *Neisseriaceae* (96-fold increase), and *Enterobacter asburiae* (183-fold increase) [51]. Tang et al. similarly reported on the enrichment of *Veillonella*, *Streptococcus*, *Lactobacillus*, and *Enterobacteriaceae*, along with the depletion of *Faecalibacterium* and *Bacteroides*. ROC curve analysis revealed that *Streptococcus* (AUC = 0.81) and *Veillonella* (AUC = 0.74) have the potential to distinguish patients with PBC from healthy controls [52]. Notably, similar microbial dysbiosis is evident in both oral and biliary tract microbiomes, suggesting that concurrent microbial alterations across multiple anatomical sites may exert synergistic effects on the pathogenesis of PBC [53].

When interpreting the above microbial alterations, several confounding factors must be considered. First, UDCA therapy itself may influence gut microbiota composition. In some studies, patients received UDCA treatment while controls did not, which may confound the distinction between the disease itself and treatment effects. Second, dietary factors were not standardized, despite ample evidence demonstrating the role of diet in shaping the gut microbiota. Additionally, disease stage (with or without cirrhosis) significantly affects the microbial profile in PBC patients. Wang et al. found significant differences in the gut microbiota between patients with cirrhotic and non-cirrhotic PBC [46]. In patients with cirrhosis, the abundance of *Faecalibacterium* and *Gemmiger*—genera with anti-inflammatory properties—was further reduced. At the same time, levels of potential opportunistic pathogens such as *Veillonella* and *Streptococcus* were relatively increased [54,55]. This suggests that gut microbial dysbiosis may become more severe as PBC progresses to cirrhosis and that alterations in specific bacterial genera could serve as microbial markers reflecting disease severity.

Finally, most studies did not exclude previous use of antibiotics, which may cause transient perturbations of the microbiota. Therefore, future research should rigorously control for these confounding factors through prospective study designs to more accurately identify PBC-specific microbial markers.

Next, we discuss the mechanisms through which the gut microbiota synergistically drives biliary injury in PBC: providing antigenic signals (e.g., sphingolipids) to activate CD1d-restricted NKT cells; regulating hepatic metabolism and immunity via metabolites (SCFAs) and bile acid signaling (FXR–FGF19); and disrupting intestinal barrier function, enabling continuous delivery of pro-inflammatory stimuli to the liver. These interconnected mechanisms form a “gut–liver–immune” positive feedback loop that amplifies disease progression.

#### 4.2.2. Molecular Modeling of the Gut Microbiome

**Figure 1:**
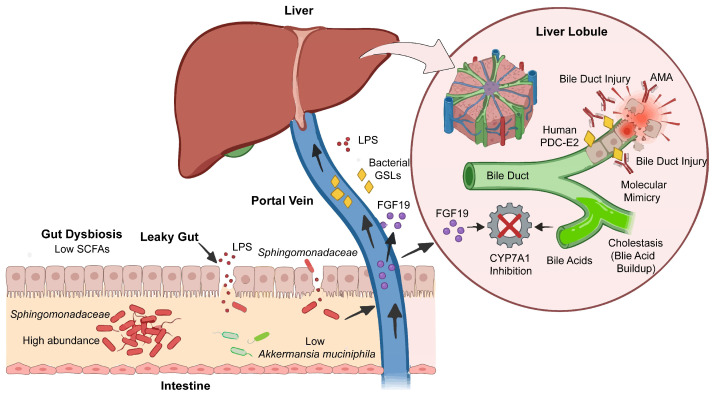
Mechanisms of gut microbiota involvement in PBC. Gut microbiota disturbances drive PBC progression via three interconnected mechanisms: ① immune activation: bacterial glycosphingolipids induce biliary epithelial injury through molecular mimicry; ② metabolic dysregulation: reduced SCFAs and FXR–FGF19 axis disruption impair bile acid metabolism, worsening cholestasis; ③ barrier dysfunction: increased intestinal permeability allows sustained translocation of bacterial products to the liver, fueling the other pathways. Together, these mechanisms form a self-amplifying positive feedback loop. Abbreviations: AMA, anti-mitochondrial antibody; CYP7A1, cholesterol 7α-hydroxylase; FGF19, fi bro blast growth factor 19; GSL, glycosphingolipid; LPS, lipopolysaccharide; PDC-E2, pyruvate dehydrogenase complex E2 subunit.

Immune dysregulation caused by gut microbiota imbalance exhibits high specificity in PBC. In the ileal mucosa of patients with PBC, *Sphingomonadaceae* (especially the genus *Sphingomonas*) and *Pseudomonas* are significantly enriched. Their cell walls contain a unique lipid component, α-glycuronyl glycolipids, which serve as natural ligands for the CD1d molecule and can be specifically recognized by the T-cell receptor of NKT cells [56]. These immune complexes, which contain bacterial GSLs and PDC-E2-like antigens, can be recognized and presented by CD1d molecules expressed on cholangiocyte surfaces. CD1d belongs to the MHC class I protein family and specializes in presenting lipid antigens. When NKT cells and Kupffer cells in hepatic sinusoids recognize antigens presented by CD1d via their receptors, they become strongly activated, triggering a cascade of inflammatory responses. Activated NKT cells and Kupffer cells release large amounts of pro-inflammatory cytokines, including Th1-type (e.g., IFN-γ) and Th2-type (e.g., IL-4) cytokines. These cytokines further recruit and activate immune cells, forming a local inflammatory storm that ultimately leads to progressive, non-suppurative destruction of the bile duct epithelium—the core pathological manifestation of chronic destructive cholangitis in PBC [57] (Figure 1).

#### 4.2.3. Gut Microbiome and Cholestasis

In PBC, gut microbiota dysbiosis disrupts bile acid metabolism through signaling axes, directly causing bile duct injury and hepatic inflammation via immune/metabolic pathways, forming a complete chain of evidence for the “gut–liver axis” pathogenesis.

The gut microbiota primarily influences cholestatic liver injury through signaling pathways associated with bile acid receptors (farnesoid X receptor, FXR; Takeda G protein-coupled receptor 5, TGR5), peroxisome proliferator-activated receptors (PPARs), and apical sodium-dependent bile acid transporter (ASBT) [58]. Among these, FXR plays a central role in microbiota–bile acid interactions as follows: the gut microbiota can suppress the synthesis of bile acid via the FXR–fibroblast growth factor 19/15 (FGF19/15) pathway [59]. In this pathway, FGF19/15 binds to fibroblast growth factor receptor 4 (FGFR4) and the co-receptor β-Klotho in the liver, thereby suppressing transcription of the cytochrome P450 family 7 subfamily A member 1 (CYP7A1) gene. The enzyme encoded by CYP7A1 is the rate-limiting enzyme in bile acid synthesis. Conversely, bile acids can also regulate the gut microbiota via FXR. In a mouse model of bile duct ligation, FXR activation reduces bacterial overgrowth and mucosal injury, thereby decreasing the incidence of bacterial translocation (Figure 1).

Although numerous studies have reported significant disturbances in the gut microbiota in patients with PBC, it has long remained unclear whether these alterations are secondary phenomena resulting from cholestasis or primary drivers of biliary injury. However, Jiang et al. transplanted fecal microbiota from treatment-naive patients with PBC into healthy mice, identifying that recipient mice developed PBC-like liver pathologies, including elevated serum ALP and total bile acids, portal inflammation, and bile duct injury. Since the recipient mice had no underlying cholestasis, these changes directly demonstrate that PBC-associated disturbances in the gut microbiota are sufficient to independently induce liver injury, rather than a mere consequence of cholestasis. Furthermore, the study found that transplanting microbiota from PBC patients into a 2OA-BSA-induced PBC mouse model significantly aggravated liver injury and bile duct inflammation. This suggests that the aberrant gut microbiota not only plays a role in disease initiation but also amplifies and sustains disease progression. Mechanistic investigations revealed that PBC microbiota may contribute to biliary injury by modulating specific immune and metabolic pathways. Liver transcriptome analysis indicated significant upregulation of immune pathways in mice colonized with PBC microbiota, including hematopoietic cell lineage, Toll-like receptor signaling, and NK cell-mediated cytotoxicity. PPAR signaling and multiple metabolic pathways were suppressed. These pathway alterations closely mirror the hepatic transcriptomic features of PBC patients, suggesting that the microbiota drives biliary injury by activating immune responses and disrupting hepatic metabolic function. Notably, this study used fecal donors who were treatment-naive for UDCA, thus effectively excluding drug-related confounding effects on microbiota composition and ensuring that the observed pathogenic effects more accurately reflected the disease state itself [57].

Taken together, current evidence strongly suggests that alterations in the gut microbiota of PBC patients are not merely secondary markers of cholestasis but primary pathogenic factors capable of driving and exacerbating bile duct injury. Of course, it must be acknowledged that existing FMT studies have largely been conducted in short-term animal models, which cannot fully recapitulate the decades-long chronic course of PBC in humans. Whether the microbiota acts as an “initiating factor” or an “amplifying factor” requires further validation in models that more closely mimic the clinical setting. Nevertheless, these findings have already provided an important theoretical rationale for gut-targeted therapeutic strategies in PBC, including probiotics, fecal microbiota transplantation, and dietary modulation.

#### 4.2.4. Dysbiotic Metabolites from the Gut Microbiota Lead to Intestinal Barrier Dysfunction

Short-chain fatty acids (SCFAs) produced by the gut microbiota have significant effects on cholangiocytes [59]. In a mouse model of α-naphthyl isothiocyanate (ANIT)-induced cholestasis, SCFAs improve bile acid metabolism by activating the FXR-FGF15-CYP7a1 signaling pathway. Concurrently, SCFAs promote the growth of *Akkermansia muciniphila*, which restores intestinal barrier function by upregulating the expression of tight junction proteins, such as ZO-1, Occludin, and Claudin-1. In patients with PBC, gut microbiota dysbiosis may reduce SCFA production, thereby diminishing their protective effects on cholangiocytes and further exacerbating cholangiocyte injury. Furthermore, fecal microbiota transplantation (FMT) studies have revealed altered metabolic functions associated with microbiota related to PBC. Compared with mice transplanted with microbiota from healthy controls, mice receiving microbiota from PBC patients exhibited significantly elevated fecal levels of 5-hydroxyindoleacetic acid (5-HIAA) and lactate (*p* < 0.05). By contrast, levels of 3-(3-hydroxyphenyl) propionate—a metabolite with anti-inflammatory and anti-resorptive properties—were significantly reduced (*p* < 0.05). These metabolic alterations were associated with enhanced inflammatory responses and downregulated lipid metabolism, and correlated significantly with changes in specific bacterial abundances: in PBC-FMT mice, the abundance of *Bacteroides congonensis* increased 4014-fold, *Dorea* sp. increased 503-fold, and beneficial bacteria such as *Bacteroides acidifaciens* were reduced to only 10% of control levels [57,60] (Figure 1).

In patients with PBC, dysbiosis of the gut microbiota triggers intestinal barrier dysfunction, thereby increasing intestinal permeability. This allows bacteria and their metabolites, such as LPS, to enter the bloodstream and reach the liver, thereby activating hepatic immune responses and damaging cholangiocytes [61]. Toll-like receptor 2 (TLR2) plays a crucial role in maintaining the integrity of the intestinal epithelial barrier [58]. Studies have shown that in TLR2 knockout mice, ZO-1 protein expression levels decrease, intestinal permeability increases, and bacterial translocation to the liver becomes more pronounced (Figure 1).

Notably, the aforementioned pathways do not function in isolation; rather, they constitute a multi-level regulatory network spanning from the gut to the liver. Disruption of the intestinal barrier permits bacterial products to translocate to the liver; these products (e.g., *Sphingomonadaceae* glycosphingolipids) activate hepatic immune responses via the CD1d–NKT cell axis. Concurrently, alterations in microbial metabolites (e.g., SCFAs) exacerbate bile acid metabolic disturbances through the FXR–FGF19 axis, further amplifying bile duct injury. These mechanisms are interlinked and collectively influence the progression of PBC (Figure 1).

Synthesizing the above discussion, a few mechanisms among the immune and metabolic pathways addressed in this review are unique to PBC. However, the value of this review lies in revealing how these general pathways are activated in a PBC-specific context: gut microbiota disturbances serve as upstream triggers, delivering sustained signals via the “gut–liver axis” to the liver, thereby driving the characteristic bile duct injury characteristic of PBC. This insight suggests that future research should focus more on PBC-specific signals originating from the gut (e.g., particular microbial antigens or metabolites), rather than solely on the downstream inflammatory pathways common to various liver diseases.

## 5. Fungal Infections Complicated with PBC: From Clinical Observation to Mechanistic Exploration

### 5.1. Clinical Evidence of Fungal Infections

In recent years, despite advances in research on gut bacteria in primary biliary cholangitis, studies on gut fungal colonization in PBC patients remain relatively scarce [62]. In the healthy human gut, the abundance of fungi is significantly lower than that of bacteria. Core fungal taxa include *Candida* species (mostly *Candida albicans*), *Saccharomyces* (mainly *Saccharomyces cerevisiae*), *Penicillium*, *Aspergillus*, *Cryptococcus*, and *Malassezia* (primarily *Malassezia restricta*) [63].

Although clinical reports of fungal infections in patients with PBC are rare, they indicate a poor prognosis. For instance, a female patient with primary biliary cholangitis and rheumatoid arthritis succumbed to liver failure after five months of co-infection with *Trichosporon mycotoxinivorans* and *Cryptococcus neoformans*, despite measures being taken to control the infection [64]. Another report indicates that a female patient receiving hormone and immunosuppressive therapy for PBC died rapidly after detection of a *Pneumocystis* spp. infection; a female patient with Sjögren’s syndrome and PBC also experienced rapid deterioration following a *mucormycosis* infection [65].

The above cases describe invasive fungal infections. According to the consensus definitions of the European Organization for Research and Treatment of Cancer/Mycoses Study Group Education and Research Consortium (EORTC/MSG), detection of pathogens from normally sterile sites or histological evidence in immunosuppressed patients confirms a diagnosis of invasive fungal disease [66]. In contrast, intestinal fungal colonization refers to the presence of fungi on mucosal surfaces without invasion into tissues, and mycobiome dysbiosis denotes alterations in the relative abundance of fungal communities with no evidence of an infectious state. These represent distinct clinical and biological entities. Furthermore, patients often received immunosuppressive therapy or presented with multiple comorbidities; thus, their adverse outcomes likely result from the interplay of multiple factors and cannot be directly attributed to fungal infection.

Notably, the cases described above involve invasive fungal infections, which belong to distinct clinical and biological categories compared to intestinal fungal colonization or alterations in fungal microbiota. However, studies on the gut mycobiome in PBC patients remain very limited; the case reports mentioned above cannot provide reliable estimates of incidence, risk factors, or causal associations. Therefore, the discussion of the mycobiome in the present study should be regarded as hypothesis-generating, and further validation is needed in large-scale cohort studies and mechanistic experiments.

### 5.2. Potential Mechanisms Underlying the Involvement of Fungi in PBC Progression

Despite limited clinical evidence, based on studies of other cholestatic liver diseases and recent advances in fungal immunology, several potential mechanisms by which fungi are involved in PBC progression can be proposed:

#### 5.2.1. Pattern Recognition of Fungal PAMPs and DAMPs

Fungal cell wall components, particularly β-glucan, provide pathogen-associated molecular patterns (PAMPs) identified by pattern recognition receptors expressed on the surfaces of intrahepatic innate immune cells, including Kupffer cells and dendritic cells [67,68]. Dectin-1 serves as the primary receptor for β-glucan. Activation of Dectin-1 triggers the Syk-CARD9 signaling pathway, induces the production of pro-inflammatory cytokines such as IL-6 and TNF-α, and promotes Th17 cell differentiation. In animal models of primary sclerosing cholangitis (PSC), activation of the Dectin-1 pathway is associated with aggravated biliary inflammation. However, direct evidence supporting a similar role in primary biliary cholangitis (PBC) is still lacking.

Dectin-1 functions as a pattern recognition receptor with dual capabilities of recognizing both fungal pathogen-associated molecular patterns (PAMPs) and damage-associated molecular patterns (DAMPs). Using a mouse colitis model, Iliev et al. demonstrated that upon recognition of fungal β-glucan (a PAMP), Dectin-1 activates the Syk-CARD9 signaling pathway. This induces the production of pro-inflammatory cytokines, such as TNF-α, and regulates the expression of the intestinal antimicrobial peptides S100A8/A9, thereby influencing the colonization of commensal bacteria like Lactobacillus. This study elucidated the classical role of Dectin-1 in fungal PAMP recognition and its regulation of the intestinal immune microenvironment [69].

Sato et al. further revealed the mechanism by which Dectin-1 senses DAMPs. In a burn mouse model, vimentin and galectin-3 released from damaged tissues acted as endogenous DAMPs. Their recognition by Dectin-1 drove excessive inflammatory responses. Compared to wild-type mice, Dectin-1 knockout mice exhibited 45–60% lower levels of inflammatory cytokines such as TNF-α and IFN-γ at the wound site, a 40% reduction in the oxidative damage marker 4-HNE, and a 30% acceleration in wound healing. Together, these two studies demonstrate that Dectin-1 plays a dual regulatory role in antifungal immunity and tissue damage repair by integrating PAMP and DAMP signals [70]. In the context of the “gut–liver axis” in PBC, this mechanism may contribute to bile duct injury and amplified inflammation due to the sensing of both fungal PAMPs and cholestasis-associated tissue DAMPs.

#### 5.2.2. Immunomodulatory Effects of Fungal Metabolites

Certain fungi produce biologically active metabolites with immunomodulatory properties. For instance, Candida hemolysin produced by *Candida albicans* can directly damage epithelial cells and activate the NLRP3 inflammasome [71,72,73]. In addition, fungal-derived prostaglandin-like molecules may modulate local inflammatory responses. Whether these metabolites contribute to pathological processes in the gut–liver axis of patients with PBC remains to be elucidated.

#### 5.2.3. The Impact of Cholestasis on Fungal Ecology

A study on PSC demonstrated that *Candida* species could be cultured from bile in approximately 12% (8/67) of patients [74], suggesting that cholestasis may alter the local microenvironment and facilitate colonization by certain fungi. Changes in the bile acid profile in stagnant bile, together with elevated biliary pressure, may confer a survival advantage to fungi, forming a vicious cycle of “cholestasis–fungal colonization–exacerbated inflammation.” This mechanism has not been systematically investigated in PBC.

### 5.3. Limitations and Future Perspectives of Fungal Research

Fungal infections worsen the prognosis of patients with PBC. Unfortunately, numerous challenges still exist in the clinical diagnosis and treatment of fungal infections in patients with PBC. First, patients with PBC and concomitant fungal infections may present without typical infectious symptoms. Furthermore, epidemiological data on the incidence, pathogen spectrum, and risk factors of fungal colonization/infection in PBC patients remain insufficient. It is challenging for clinicians to initially associate non-specific symptoms with life-threatening fungal infections, resulting in significant delays in recognition and diagnosis. Second, blood cultures have low detection rates for fungi (especially *Candida*), and routine imaging lacks specificity. Diagnosis typically relies on procedures like bronchoscopy, aspiration, or biopsy. However, patients with advanced PBC often cannot tolerate these procedures due to coagulation disorders, thrombocytopenia, and poor general condition. Furthermore, the hepatotoxicity of effective antifungal drugs conflicts with the existing liver damage in PBC. Simultaneously, the need to reduce immunosuppression for infection control conflicts with the requirement to suppress PBC’s autoimmune activity. This creates a “therapeutic paradox” in clinical decision-making, ultimately leading to an extremely poor prognosis. Given that the underlying mechanisms of fungal infection remain unclear, animal models that recapitulate fungal infection in the context of PBC are still lacking.

Therefore, clinicians must maintain a high index of suspicion and initiate early preventive, diagnostic, and therapeutic strategies to treat fungal infections. Meanwhile, there is an urgent need for metagenomic studies of the gut mycobiome in patients with PBC, pharmacokinetic and safety studies on the use of antifungal agents in this population, the establishment of PBC-specific animal models of fungal infection, and further well-designed in vitro and in vivo studies clarifying the impact of cholestasis on fungal colonization. These efforts will help improve the diagnosis, treatment, and prognosis of PBC in patients with concurrent fungal infections.

## 6. Shortcomings and Prospects

Research into the infection status and pathogenesis of PBC patients—whether bacterial or fungal—reveals significant and interrelated knowledge gaps at both the clinical and basic science levels. In clinical studies of bacterial infections, high-quality evidence regarding the incidence and prognosis of specific bacterial infections at different stages of PBC is scarce. Studies on large-scale cohorts are lacking, and existing data are often confounded with those from patients with advanced cirrhosis. Furthermore, no consensus has been reached on strategies to prevent infection (such as antibiotic prophylaxis) or management approaches specific to PBC. Research on drug interactions and dose adjustments between UDCA and novel agents (e.g., obeticholic acid) and commonly used antibiotics is also limited, resulting in scant guidance for clinical practice. At the mechanistic level, most studies remain confined to broad descriptions of “immune dysfunction.” Further exploration is needed into how PBC-specific immune dysregulation—such as altered bile acid receptor signaling—impacts the antimicrobial functions of neutrophils and macrophages (Table 2).

Furthermore, epidemiological data on fungal infections in PBC are extremely scarce, with fundamental information regarding incidence rates, pathogen spectrum, and specific risk factors remaining largely absent. Currently, there are no risk-stratified screening or diagnostic pathways tailored to the PBC population. Additionally, studies on the hepatotoxicity and pharmacokinetics of antifungal drugs in patients with PBC are lacking, resulting in treatment decisions being largely based on empirical evidence. At the basic research level, the molecular and immunological mechanisms underlying fungal colonization, translocation, and pathogenicity in the context of PBC remain poorly understood. Notably, the potential impact of cholestasis itself on bile and gut fungal ecology—a potentially key mechanistic pathway—has not been systematically investigated. Furthermore, there is an urgent need for reliable animal or cellular models that can faithfully replicate fungal infection in the context of PBC (Table 2).

To address these gaps, future research should adopt a multifaceted and comprehensive approach. At the clinical level, there is an urgent need for large-scale, prospective, multicenter cohort studies to establish accurate epidemiological data on bacterial and fungal infections across all stages of PBC. These data should be distinguished from other causes of cirrhosis. Such studies should provide evidence-based guidelines for infection prevention, risk-stratified screening, and tailored management in PBC. Concurrent pharmacology studies should focus on drug interactions and optimal dosing strategies involving ursodeoxycholic acid, novel therapeutics, and antimicrobials. At the level of basic science, efforts must extend beyond descriptive immunology to elucidate the specific microscopic mechanisms by which PBC-associated immune dysregulation and cholestatic injury impair innate immune cell function and reshape microbial and fungal communities, particularly along the gut–liver axis. This will necessitate the development of reliable preclinical models that replicate the complex infection patterns observed in PBC. Furthermore, specialized pharmacokinetic and hepatotoxicity studies of antifungal agents in patients with PBC are critical for guiding safe and effective therapies (Table 2).

Ultimately, integrating deep clinical phenotyping with mechanistic insights is crucial for developing targeted strategies to prevent, diagnose early, and improve outcomes in PBC infections.

## 7. Conclusions

In summary, existing evidence indicates a close association between bacterial infections and the development and progression of PBC. *Escherichia coli* is the best-characterized pathogen with the most robust evidence for activating cross-immune responses through molecular mimicry. *Novosphingobium aromaticivorans* and other commensal bacteria have pathogenic potential in animal models; however, their direct roles in humans remain to be verified. Intestinal dysbiosis is prevalent in patients with PBC, and studies on fecal microbiota transplantation suggest its potential to influence the progression of disease. However, distinguishing whether microbiota alterations are a cause or consequence of the disease remains a major challenge. In contrast, the role of fungi in PBC is still in the early exploratory stage. Clinical case reports suggest an association with poor prognosis, but mechanistic studies on this subject are almost nonexistent. Future research urgently requires large-scale, prospective longitudinal cohort studies integrating culturomics, metabolomics, and mono-colonization experiments in germ-free animals to evaluate the potential of interventions such as targeted probiotics and FXR agonists in regulating the microbiota–immune axis, as well as to establish risk stratification and screening pathways for fungal infections and safety monitor protocols for antifungal agents in PBC patients. Elucidating this complex microbial network will not only deepen the understanding of PBC pathogenesis but also provide a theoretical basis for developing novel diagnostic tools and targeted therapeutic strategies.

## Figures and Tables

**Table 2:** Research gaps and clinical significance of bacterial and fungal infections in PBC.

Clinical Domain	Key Challenges/Current Dilemmas	Clinical Recommendations/Practical Considerations
Diagnostic Challenges	Cholestasis itself (elevated ALP/GGT) may mask laboratory manifestations of infection, and potentially be misattributed to PBC disease activity. Immunosuppressive therapy (e.g., corticosteroids for overlap syndromes) can attenuate fever and other signs of infection, leading to delayed diagnosis. Distinguishing fungal colonization (common in advanced liver disease) from invasive infection lacks PBC-specific criteria. Whether the pathogen spectrum of infections in PBC patients is distinct remains unclear, leaving empirical anti-infective therapy without a solid foundation.	Maintain a high index of suspicion: Actively screen for infection in PBC patients, especially those on immunosuppressive therapy, presenting with unexplained fluctuations in liver function, worsening fatigue, or systemic symptoms. Lower the threshold for diagnostic workup: Incorporate blood cultures, fungal G/GM tests, and imaging into the differential diagnosis algorithm. Reference EORTC/MSG criteria: In immunosuppressed PBC patients, pathogen detection from normally sterile sites and histological evidence should be considered indicative of invasive infection, rather than colonization. Consider developing a clinical warning score or flowchart for diagnosis of infection in PBC patients.
Therapeutic Confounders	The impact of core PBC therapies (UDCA, OCA) on gut microbiota and infection risk remains unclear. Immunosuppressants (corticosteroids, mycophenolate, etc.) serve both as risk factors for infection and may mask existing infections. Anti-infective therapy (antibiotics/antifungals) may interact with PBC medications or further disrupt gut–liver axis homeostasis. Evidence is lacking on whether UDCA/OCA dosage should be adjusted or suspended during active infection.	Individualized risk assessment: Evaluate infection risk comprehensively (liver disease stage, age, and comorbidities) before initiating immunosuppressive therapy. Multidisciplinary collaboration: For PBC complicated by complex infections, involve hepatology, infectious disease, and clinical pharmacy specialists in treatment decisions. Monitor drug interactions: When using azole antifungals, monitor liver function and, if possible, PBC medication in blood levels. Reassess the benefit–risk ratio of PBC baseline therapy after infection control.
Prognostic Considerations	The question of whether infectious events independently accelerate PBC disease progression (fibrosis, cirrhotic decompensation, and liver failure) lacks longitudinal data. Risk stratification models are missing to identify which PBC subgroups (e.g., advanced fibrosis, immunosuppressed, and those with coexisting autoimmune diseases) face the highest risk of infection. Case reports suggest invasive fungal infections may be associated with rapid clinical deterioration, but causality remains unconfirmed. Conceptually, whether infection should be considered part of PBC disease activity or an independent complication remains ambiguous.	Identify high-risk populations: Prioritize enhanced monitoring for patients with advanced PBC, those on immunosuppressive therapy, those with hypoalbuminemia, and those with a history of infection. Integrate infections into overall management: Document infectious events as potential prognostic indicators during routine PBC follow-up. Patient education: Inform patients with PBC on immunosuppressive therapy about risks of infection and indications for early medical consultation. The occurrence of an infectious event should prompt clinicians to reassess the patient’s PBC disease stage and treatment plan.
Translational Research Needs	Basic science discoveries (e.g., IL-15 pathway and cGAS-STING pathway) have yet to be translated into clinical infection risk predictors or intervention targets. Multidimensional PBC patient stratification models integrating clinical phenotypes, immune status, and microbiome data are lacking.	Bidirectional clinical-basic translation: Use clinically observed high-risk PBC subgroups as entry points for mechanistic research. Establish clinical biorepositories: Collect serum, stool, and tissue samples from PBC patients with and without infections for subsequent validation studies. Explore microbiome modulation (probiotics, fecal microbiota transplantation) as an adjunctive therapy in PBC, while simultaneously monitoring risk of infection.

## Data Availability

No new data were created or analyzed in this study. Data sharing is not applicable to this article.

## Abbreviations

The following abbreviations are used in this manuscript:

PBC	primary biliary cholangitis
ALP	alkaline phosphatase
GGT	γ-glutamyl transpeptidase
HBRV	human β-retrovirus
AMAs	anti-mitochondrial antibodies
BECs	bile duct epithelial cells
S1PR2	sphingosine-1-phosphate receptor 2
HSCs	hepatic stellate cells
MAPK	mitogen-activated protein kinase
YAP	Yes-associated protein
IL-17	interleukin-17
TNF-α	tumor necrosis factor-alpha
JCAD	junctional adhesion molecule
LATS1/2	large tumor suppressor 1/2
HCC	hepatocellular carcinoma
UTIs	urinary tract infections
PDC-E2	pyruvate dehydrogenase complex E2 subunit pyruvate dehydrogenase complex E2 subunit
TCRβ	T-cell receptor beta
*E. coli*	*Escherichia coli*
NKTs	natural killer T cells
Tfhs	follicular helper T cells
PD-1	programmed cell death protein 1
CD40L	CD40 ligand
ICOS	inducible co-stimulatory molecule
TLR	Toll-like receptor
LPS	lipopolysaccharide
IFN-γ	interferon-γ
SCFAs	short-chain fatty acids
FXR	farnesoid X receptor
TGR5	Takeda G protein-coupled receptor5
PPARs	peroxisome proliferator-activated receptors
ASBT	apical sodium-dependent bile acid transporter
FGF19/15	fibroblast growth factor 19/15
FGFR4	fibroblast growth factor receptor 4
CYP7A1	cytochrome P450 family 7 subfamily A member 1
FMT	fecal microbiota transplantation
ANIT	α-naphthyl isothiocyanate
5-HIAA	5-hydroxyindoleacetic acid
TLR2	Toll-like receptor 2
PAMPs	pathogen-associated molecular patterns
DAMPs	damage-associated molecular patterns
PSC	primary sclerosing cholangitis
